# Analyzing Taphonomic Deformation of Ankylosaur Skulls Using Retrodeformation and Finite Element Analysis

**DOI:** 10.1371/journal.pone.0039323

**Published:** 2012-06-22

**Authors:** Victoria M. Arbour, Philip J. Currie

**Affiliations:** Department of Biological Sciences, University of Alberta, Edmonton, Alberta, Canada; University of Pennsylvania, United States of America

## Abstract

Taphonomic deformation can make the interpretation of vertebrate fossil morphology difficult. The effects of taphonomic deformation are investigated in two ankylosaurid dinosaur taxa, *Euoplocephalus tutus* (to investigate effects on our understanding of intraspecific variation) and *Minotaurasaurus ramachandrani* (to investigate the validity of this genus). The ratio of orbit maximum rostrocaudal length to perpendicular height is used as a strain ellipse, which can be used to determine if ankylosaur skull fossils have been dorsoventrally compacted during fossilization and diagenesis. The software program Geomagic is used to retrodeform three-dimensional (3D) digital models of the ankylosaur skulls. The effects of sediment compaction are modeled using finite element analysis, and the resulting strain distributions are compared with the retrodeformed models as a test of the retrodeformation method. Taphonomic deformation can account for a large amount of intraspecific variation in *Euoplocephalus*, but finite element analysis and retrodeformation of *Minotaurasaurus* shows that many of its diagnostic features are unlikely to result from deformation.

## Introduction

Variation among specimens referred to a single fossil taxon can originate from several biological sources, such as ontogeny, sexual dimorphism, and individual variation, but taphonomy can also be a source of morphological variation in fossils. During fossilization and diagenesis, bones can become deformed, and this deformation can lead to difficulties in understanding taxonomic variation, phylogenetic relationships, and functional morphology [1], [2], [3], [4]. Understanding the effects of taphonomic deformation on bones is therefore important for interpreting morphological variation.

Fossils can become distorted from the effects of brittle or plastic deformation (or both). In geological terms, brittle deformation results in fractures, joints, and faults, and plastic deformation results in folds. Whether or not a fossil undergoes brittle or plastic deformation is dependent on the temperature, confining pressure, and strain rate it experiences. Brittle deformation occurs at low temperatures, low confining pressures, and high strain rates; plastic deformation occurs at high temperatures, high confining pressures, and low strain rates. Many fossils undergo brittle deformation prior to burial, cracking and fracturing during transport, and brittle deformation can occur during diagenesis as well, such as if a fossil is faulted. Plastic deformation of a fossil is more likely to occur during fossilization and diagenesis, during which time bone can act like a ductile material. Fossils rarely survive more than a single phase of plastic deformation, and as such, identifiable but plastically distorted fossils typically have a simple deformation history [5]. Not all fossils in a single bedding plane may deform homogeneously, and not all elements within a single specimen will necessarily deform homogeneously [5]. The orientation of a specimen within the sediment will also affect how the specimen deforms [6].

The goal of this study is to introduce some techniques for understanding three-dimensional (3D) plastic deformation in ankylosaurid dinosaur skulls. First, skulls of extant vertebrates were examined to determine if the shape of the orbit can be used as an indicator for whether or not plastic deformation has occurred. If the periorbital rims of a variety of extant vertebrates are generally circular, then fossil skulls with elliptical orbits have probably undergone some amount of plastic deformation. Retrodeformation and finite element analysis were then used as tools for understanding what parts of an ankylosaur skull are most likely to undergo deformation and therefore least likely to be phylogenetically useful. This information can then be used to enhance the quality of cranial characters used in phylogenetic analyses. No attempt was made to undistort taphonomically distorted skulls into their original shape, as there are few features on the skull to act as constraints guiding the decisions in retrodeforming ankylosaur skulls. Retrodeforming an ankylosaur skull with the goal of restoring its true shape would be highly subjective. Instead, the focus of this study is on understanding which morphological features on an ankylosaur skull are most likely to become taphonomically deformed.

The software program Geomagic [7] is used to investigate potential effects of deformation by modifying digital models of ankylosaur skulls. It can be used to restore symmetry to a skull, and to measure the amount of shape change in various models of the same structure. Finite element analysis (FEA) can be used to investigate the way in which we might expect a fossil to have deformed under a variety of geological forces. FEA has been used to investigate the effects of biologically-induced forces in extant and extinct vertebrates [8], [9], [10]. However, FEA has not been used to investigate the effects of geological forces on vertebrate fossils, such as sediment compaction and diagenesis. In this paper, the retrodeformation analyses represent the subtraction of deformation from a skull, and the finite element analyses represent the addition of deformation to a skull. If the same parts of the skull undergo shape change during both retrodeformation and FEA, then these parts of the skull are most likely to experience deformation during fossilization and diagenesis.

**Figure 1:**
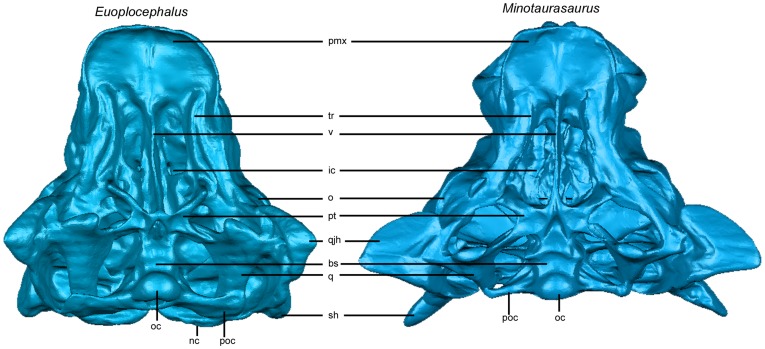
Comparison of AMNH 5405 (*Euoplocephalus*) and INBR 21004 (*Minotaurasaurus*) in ventral view. Specimens scaled to same premaxilla-occipital condyle length. Abbreviations: bs – basisphenoid, ic – internal choana, nc – nuchal crest, o – orbit, oc – occipital condyle, pmx – premaxilla, poc – paroccipital process, pt – pterygoid, q – quadrate, qjh – quadratojugal horn, sh – squamosal horn, tr – tooth row, v – vomer.

This study examines two cases where understanding deformation can be used to better interpret ankylosaur cranial morphology: 1) intraspecific variation in *Euoplocephalus tutus* Lambe, 1910 [11], and 2) the taxonomic validity of *Minotaurasaurus ramachandrani* Miles and Miles, 2009 [12]. *Euoplocephalus* (Fig. 1) is the best represented ankylosaurid from the Late Cretaceous of North America, and more than 15 skulls have been referred to this genus. Coombs [13] synonymized four taxa within *Euoplocephalus tutus*: *Dyoplosaurus acutosquameus* Parks, 1924 [14], *Scolosaurus cutleri* Nopcsa, 1928 [15], and *Anodontosaurus lambei* Sternberg, 1929 [16]. Arbour et al. [17] recognized *Dyoplosaurus* as a distinct taxon, a result supported by an ankylosaur phylogenetic analysis by Thompson et al. [18]. Penkalski [19] noted a great deal of variation among skulls referred to *Euoplocephalus*, and identified sexual dimorphism, ontogeny, and individual differences as the sources for much of this variation, in addition to potential taxonomic differences. Many of the distinctive features of individual specimens noted by Penkalski [19] are unlikely to change during deformation, because they represent quantities or surface texture (e.g. surface texture of cranial sculpturing, number of osteoderms in the nuchal crest). However, some, such as the erectness of the squamosal horns, may be affected by dorsoventral compaction. Retrodeformation of two *Euoplocephalus* skulls (AMNH 5405 and UALVP 31) will highlight the changes that can occur during crushing, and can be used to identify areas of the skull that are most likely to change and therefore be less taxonomically informative. For example, if the erectness of the squamosal horns changes with retrodeformation, or if there is high strain in this area after FEA, then the erectness of the squamosal horn may be affected by dorsoventral compaction.

The second case study examines the taxonomic validity of *Minotaurasaurus* (Fig. 1), known from a single specimen of unknown provenance, but likely from the Gobi Desert of Mongolia or China [12]. This taxon bears a strong overall resemblance to *Saichania chulsanensis* Maryańska, 1977 [20], *Tarchia gigantea* Maryańska, 1977 [20], and *Tianzhenosaurus youngi* Pang and Cheng, 1998 [21], although the most recent phylogenetic analysis of the Ankylosauria [18] found a close relationship between *Minotaurasaurus ramachandrani* and *Pinacosaurus grangeri* Gilmore, 1933 [22] (but not *Pinacosaurus mephistocephalus* Godefroit, Pereda Suberbiola, Li, and Dong, 1999 [23]). Although the holotype of *Minotaurasaurus* does not appear obviously taphonomically distorted, it has a much lower, flatter profile compared to ankylosaurs such as *Euoplocephalus*. Additionally, several features are described by Miles and Miles [12] as being flatter or more dorsoventrally compressed compared to other taxa, such as the orientation of the pterygoid, the articular surface of the quadrate, the pterygoid-quadrate contact, and the angle of projection of the quadratojugal horn. If the pterygoid, quadrate, and quadratojugal horn undergo more shape change than other portions of the skull during retrodeformation and FEA, then these features are most likely the result of dorsoventral compaction and the diagnosis of *Minotaurasaurus* should be revised.

## Methods

### The Orbit as a Strain Ellipse

**Figure 2:**
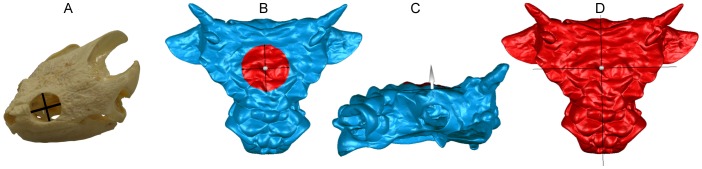
Measuring orbit shape, and deforming digital models in Geomagic. A) Two dimensions were measured for each orbit, the maximum rostrocaudal length, and the perpendicular height, shown here on TMP 1999.58.79, *Chelydra serpentina*. B) To retrodeform digital skull models in Geomagic, the “Deform Region” tool is selected and placed at the midline of the skull, between the orbits. C) The arrow is adjusted into the desired position, in this case, pointing dorsally. D) The tool is then expanded to encompass the entire skull.

In order to identify crushed ankylosaur skulls, it is necessary to identify a feature on the skull that has a particular shape or symmetry in the undeformed state. The change in size and shape that a body undergoes during deformation is known as strain [24]. Strain can be represented by a strain ellipsoid (or strain ellipse, for plane strain). The shape of a strain ellipse is described by determining the ratio of the principal axes, the ellipticity (R). The strain ellipse is useful for studies of retrodeformation because it indicates the magnitude and orientation of deformation. Srivastava and Shah [25] noted that circular objects such as crinoid stems deform into ellipses. A possible strain ellipse in vertebrate skulls could be the orbit, but the shape of a normal, undeformed orbit needs to be determined. Orbits of extant vertebrate skulls in the TMP, UALVP, and UAMZ collections (institutional abbreviations in Table 1) were measured to determine the range of shape variation within and among taxa. The greatest dimension of the periorbital rim (approximately the rostrocaudal length of the orbit), and the perpendicular dimension (which together are the major and minor axes of the ellipse) were measured using digital calipers placed flush with the bone surface (Fig. 2). The sample includes mammals, turtles, squamates, crocodilians, and birds. Birds and squamates are poorly represented in this sample because most do not have continuous periorbital rims, making it difficult to accurately measure the maximum rostrocaudal lengths of the orbits. The sample is also biased towards large mammals because these were easier to measure accurately and more were available for study. The same measurements were collected for a variety of ankylosaurid taxa. Measurements for two ankylosaur skulls (AMNH 5214 and AMNH 5404) were obtained using photographs and the software program ImageJ [26] because these two specimens are mounted behind glass; all other specimens were measured directly from real or cast specimens.

**Table 1:** Institutional abbreviations and locations.

Abbreviation	Institution	Location
**AMNH**	American Museum of Natural History	New York, New York, USA
**BMNH**	Natural History Museum	London, UK
**BXGM**	Benxi Geological Museum	Benxi, Liaoning, China
**CMN**	Canadian Museum of Nature	Ottawa, Ontario, Canada
**INBR**	Victor Valley Museum	Apple Valley, California, USA
**IVPP**	Institute for Vertebrate Paleontology and Paleoanthropology	Beijing, China
**MOR**	Museum of the Rockies	Bozeman, Montana, USA
**MPC**	Mongolian Paleontological Centre	Ulaanbaatar, Mongolia
**PIN**	Paleontological Institute	Moscow, Russia
**ROM**	Royal Ontario Museum	Toronto, Ontario, Canada
**SMP**	State Museum of Pennsylvania	Harrisburg, Pennsylvania, USA
**TMP**	Royal Tyrrell Museum of Palaeontology	Drumheller, Alberta, Canada
**UALVP**	University of Alberta Laboratory for Vertebrate Paleontology	Edmonton, Alberta, Canada
**UAMZ**	University of Alberta Museum of Zoology	Edmonton, Alberta, Canada
**USNM**	Smithsonian Museum of Natural History	Washington, DC, USA
**ZPAL**	Zoological Institute of Paleobiology, Polish Academy of Sciences	Warsaw, Poland

### 3D Model Creation

Three ankylosaur skulls were converted into 3D digital models from computed tomography (CT) scans. UALVP 31 (*Euoplocephalus*) was CT scanned at the University of Alberta Hospital ABACUS Facility. CT scans of the holotype of *Minotaurasaurus* (INBR 21004) were provided by V.S. Ramachandran (University of California San Diego). L. Witmer (Ohio University Heritage College of Osteopathic Medicine) provided CT scans of AMNH 5405, (*Euoplocephalus*), which were originally published in Witmer and Ridgely [27]. New 3D models of AMNH 5405, INBR 21004, and UALVP 31 were created from the CT data using the segmentation tools in the software program Mimics [28]. Rock matrix was digitally removed from the nasal cavities and endocranial spaces, and cracks in the bones were filled. These models were then exported as surface stereolithography (.stl) files for importing into Geomagic.

### 3D Retrodeformation in Geomagic

To investigate the effects of dorsoventral compaction, the models of *Minotaurasaurus* and two *Euoplocephalus* specimens (AMNH 5405 and UALVP 31) were imported into the software program Geomagic and retrodeformed using the Deform Region tool (Fig. 2). The tool was placed at the midline on the dorsal surface of each skull, at the midlength of the orbits. The skull was then ‘pulled’ and ‘pushed’ in the dorsoventral plane using the distance criterion tool.

### Finite Element Analysis of Taphonomic Deformation

The AMNH 5405 and INBR21004 stereolithography files were reimported into Mimics in order to create volume meshes for finite element analyses, in order to test the effects of potential geological forces on ankylosaur skulls. These volume meshes were exported as Nastran (.nas) files and imported into the software program Strand7 [29]. The models were given the material properties of compact bone (Poisson’s ratio  = 0.4, and Young’s modulus  =  8 10^9^ GPa; see [30]). Deformation could also occur after permineralization, but the material properties of the average fossil bone from the Dinosaur Park Formation (from which both specimens of *Euoplocephalus* were recovered) are unknown, and the provenance of the holotype of *Minotaurasaurus* is unknown. Finally, each of the models were put through five different analyses (Table 2) approximating dorsoventral compaction, and analyzed using the linear static solver in Strand7, solving for stress, strain, and displacement. Each analysis models the effects of dorsoventral compaction on an ankylosaur skull that is resting on a horizontal surface with the dorsal side up, with forces acting downwards in the vertical direction. These conditions are meant to approximate the forces acting on a skull during burial and sediment compaction: ankylosaur skulls are wider than tall and more likely to come to rest on a flat surface either right-side-up or upside-down. As the skull becomes buried, the weight of sediment will exert downwards, vertical forces on the skull. The number of nodes with constraints and/or forces applied is increased in each analysis, to create a number of potential scenarios mimicking dorsoventral compaction. It should be noted that the absolute values of force used are irrelevant for this test, because it is only the distribution of strain, and not the value of absolute strain, that is of interest.

**Table 2:** Summary of force and constraint parameters in five finite element tests simulating taphonomic deformation of AMNH 5405 and INBR 21004.

	Constraint Location	Force Location and Direction
Test 1	On the rostrolateral edges of the premaxilla, and on the medial endof each quadrate head.	On the dorsal surface at the midline between the orbits, ventrally directed.
Test 2	On the rostrolateral edges of the premaxilla, on the medial endof each quadrate head, and on the ventrolateral tip ofthe quadratojugal horns.	On the dorsal surface at the midline between the orbits, ventrally directed.
Test 3	As for Test 2.	On the dorsal surface at the midline between the orbits, ventrolaterally directed.
Test 4	As for Test 2.	On the dorsal surface at the midline between the orbits, and at the midline near the rostral end of the maxilla, ventrally directed.
Test 5	As for Test 2.	On the dorsal surface at the midline between the orbits, at the midline near the rostral end of the maxilla, and at the distal tip of each squamosal horn, ventrally directed.

## Results

### Results of Orbit Shape Measurements

**Figure 3:**
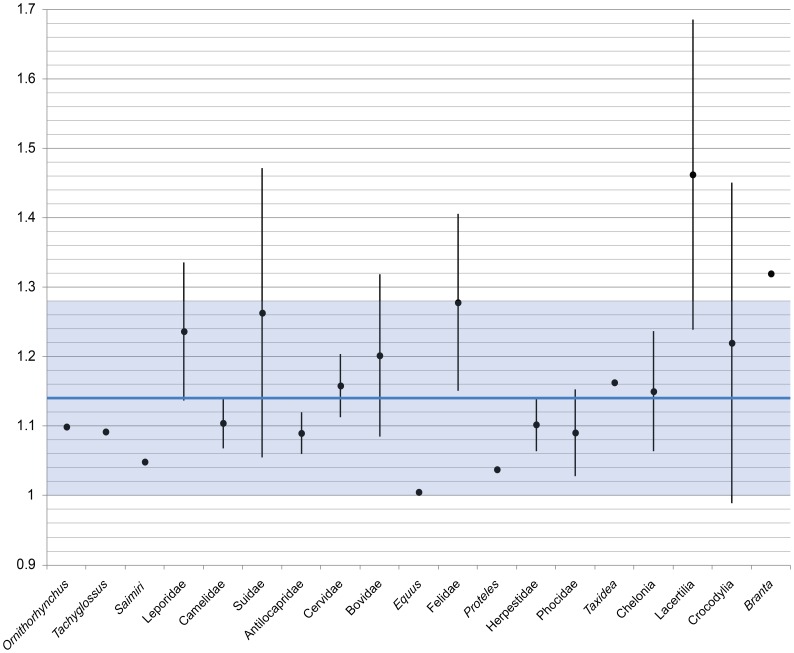
Results of orbit shape measurements for extant taxa. The mean ratio for each taxon is represented by the black circle, and the standard deviation by the vertical line. The blue horizontal line shows the mean ratio for all taxa except crocodilians and lizards, and the light blue box represents the standard deviation. The mean orbit ratio is 1.14±0.14 (n  = 96).

**Figure 4:**
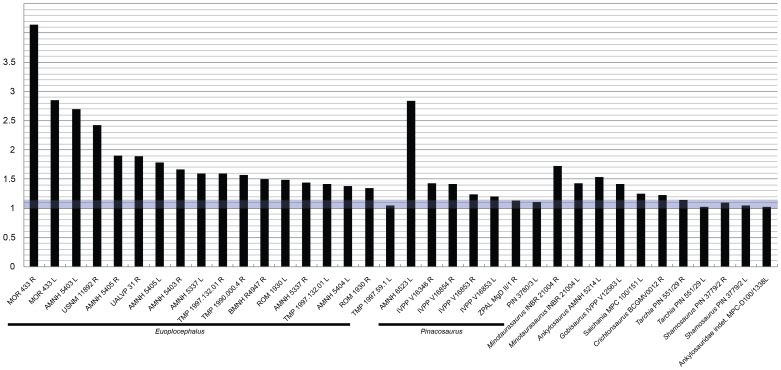
Results of orbit shape measurements for ankylosaurs. An R or L after the specimen number denotes the right or left orbit, respectively. The light blue box represents the mean orbit ratio ± one standard deviation for extant taxa (1.14±0.14).

Orbit shape measurements of extant taxa (Table 3, Fig. 3) have a mean rostrocaudal length:dorsoventral height ratio of 1.14±0.14; archosaurs have higher orbit ratios compared to mammals. Few specimens of ankylosaurs (Table 4, Fig. 4) have an orbit ratio below 1.28. Several ankylosaur specimens (AMNH 5403, MOR 433) have noticeably different orbit ratios for the left and right orbits.

**Table 3:** Orbit rostrocaudal length:dorsoventral height ratios of extant taxa.

Family	Species	Mean ± SD	Number of Specimens
Ornithorhynchidae	*Ornithorhynchus anatinus*	1.10	**1**
Tachyglossidae	*Tachyglossus aculeatus*	1.09	**1**
Cebidae	*Saimiri* sp.	1.05	**1**
Leporidae		1.24±0.10	**2**
	*Lepus americanus*	1.17	1
	*Oryctolagus cuniculus*	1.31	1
Camelidae	*Lama glama*	1.10±0.04	**2**
Suidae		1.26±0.21	**5**
	*Babyrousa babyrussa*	1.54	1
	*Pecari tajacu*	1.05±0.06	2
	*Phacochoerus aethiopicus*	1.13	1
	*Potamochoerus porcus*	1.16	1
Cervidae		1.16±0.05	**25**
	*Alces alces*	1.08±0.05	10
	*Cervus canadensis*	1.10	1
	*Muntiacus* sp.	1.07	1
	*Odocoileus hemionus*	1.05±0.04	2
	*Odocoileus virgianus*	1.07±0.02	4
	*Rangifer tarandus*	1.09±0.06	7
Antilocapridae	*Antilocapra americana*	1.09±0.03	**4**
Bovidae		1.20±0.12	**20**
	*Bison bison*	1.02	1
	*Bos taurus*	1.07±0.16	4
	*Damaliscus hunteri*	1.17	1
	*Kobus ellipsiprymnus defassa*	1.02	1
	*Oreamnos americanus*	1.13±0.03	8
	*Ovibos moschatus*	1.04±0.01	3
	*Ovis canadensis*	1.46	1
	*Syncerus caffer*	1.01	1
Equidae	*Equus caballus*	1.01	**1**
Felidae		1.28±0.13	**8**
	*Felis concolor*	1.25±0.10	6
	*Felis pardus*	1.18	1
	*Panthera tigris*	1.51	1
Hyaenadae	*Proteles cristata*	1.03	**1**
Herpestidae		1.10±0.04	**4**
	*Cynictis penicillata*	1.09±0.02	2
	*Galerella pulverulenta*	1.11±0.06	2
Phocidae		1.09±0.06	**5**
	*Erignathus barbatus*	1.11±0.06	2
	*Halichoerus grypus*	1.16	1
	*Pusa hispida*	1.04±0.01	2
Mustelidae	*Taxidea taxus*	1.16	**1**
Chelydridae		1.11±0.02	**4**
	*Chelydra serpentina*	1.11±0.02	3
	*Macrochelys temminckii*	1.13	1
Emydidae	*Terrapene carolina*	1.30	**1**
Helodermatidae	*Heloderma suspectum*	1.09	**1**
Varanidae	*Varanus* spp.	1.56±0.09	**4**
Gavialidae	*Tomistoma schlegelii*	1.10	**1**
Alligatoridae		1.32±0.34	**2**
	*Melanosuchus niger*	1.56	1
	*Paleosuchus trigonatus*	1.08	1
Crocodylidae	*Crocodylus niloticus*	1.13	**1**
Anatidae	*Branta canadensis*	1.32	**1**
**Total**		**1.15±0.14**	**96**

**Table 4:** Orbit rostrocaudal length:dorsoventral height ratios of ankylosaurid specimens.

Taxon	Specimen Number	Right OrbitWidth: Height	Left OrbitWidth: Height
*Ankylosauridae indet.*	MPC-D100/1338		1.03
*Ankylosaurus magniventris*	AMNH 5214		^A^1.53
*Crichtonsaurus benxiensis*	BXGMV0012 R	B1.23	
*Euoplocephalus tutus*	AMNH 5337	1.44	1.59
	AMNH 5403	1.663	2.69
	AMNH 5404		^C^1.38
	AMNH 5405	1.90	1.18
	BMNH R4947	1.50	
	MOR 433	4.15	2.85
	ROM 1930	1.35	1.49
	TMP 1997.132.01	1.59	1.42
	TMP 1997.59.1		1.05
	UALVP 31	1.89	2.13
	USNM 11892	2.42	
*Pinacosaurus grangeri*	AMNH 6523		2.84
	IVPP V16346	1.43	
	IVPP V16853	1.24	1.20
	IVPP V16854	1.42	
	PIN 3780/3		1.10
	ZPAL MgD II/1	1.13	
*Gobisaurus domoculus*	IVPP V12563	D1.57	1.41
*Minotaurasaurus ramachandrani*	INBR 21004	E1.72	1.43
*Saichania chulsanensis*	MPC 100/151		F1.25
*Shamosaurus scutatus*	PIN 3779/2	1.09	1.05
*Tarchia gigantea*	PIN 551/29	1.14	1.02

A,C AMNH 5214 and AMNH 5404 are mounted behind glass, but because the ratio does not require absolute values, the ratio can be determined using a photograph orthogonal to the orbit and the software program ImageJ [26].

B Measured from cast UALVP 52015.

D Measured from cast TMP 1990.000.0004.

E Measured from cast UALVP 49402.

F Measured from cast mounted with MPC 100/1305. MPC-D100/1338 is an indeterminate ankylosaurid from the Nemegt Formation of Mongolia.

### Retrodeforming Ankylosaur Skulls

**Figure 5:**
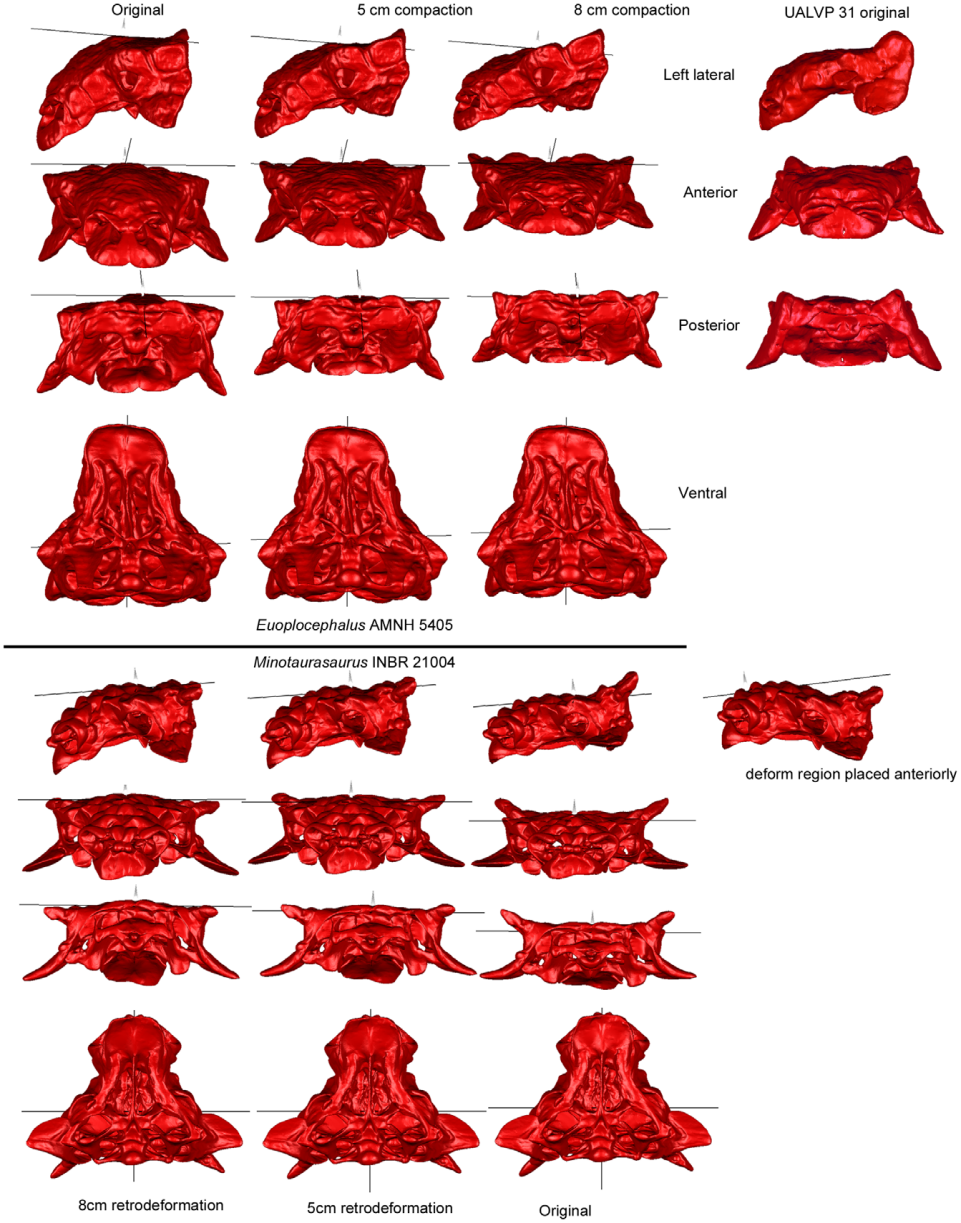
Results of deformation and retrodeformation of models using Geomagic. The top half of the image shows AMNH 5405 with (from left to right) no compression, 5 cm compression, and 8 cm compression; the rightmost column shows the original UALVP 31 skull for comparison. The bottom half of the image shows INBR 21004 with (from left to right) 8 cm retrodeformation, 5 cm retrodeformation, and no retrodeformation.

The original AMNH 5405 *Euoplocephalus* skull is bilaterally asymmetrical, but the arched profile in lateral view suggests that the skull has not been dorsoventrally compacted. Surprisingly, the orbit ratios (left 1.78, right 1.9) are higher than what would be expected if the skull was not crushed at all (Fig. 4), and are similar to that for UALVP 31 (1.89). Deforming the digital skull in Geomagic resulted in less dorsoventral height, more upright squamosal horns relative to the rest of the skull, and more laterally projecting quadratojugal horns (Fig. 5). The nuchal crest became more dorsally prominent in rostral view. The ventral edge of the paroccipital process became more horizontally oriented. Changes were minimal on the ventral surface of the skull. Dorsoventrally compressing AMNH 5405 by 8 cm in Geomagic resulted in a shape similar to that seen in UALVP 31, suggesting that the differences between these two specimens may be due to taphonomic changes.

The *Minotaurasaurus* skull (INBR21004) is low and flat in lateral view and is nearly symmetrical. The orbit ratios are 1.72 (right) and 1.43 (left), which is slightly higher than what would be expected based on the survey of extant skulls. The orbits are also teardrop-shaped, which suggests that the skull may have been dorsoventrally compressed. Retrodeforming the skull in Geomagic resulted in an arched rostrum similar to that of AMNH 5405, more horizontally projecting squamosal horns, and more ventrally projecting quadratojugal horns (Fig. 5). The dorsal margins of the paroccipital processes and the supraoccipital became curved. There were few changes to the ventral surface of the skull.

### Finite Element Analysis of Taphonomic Deformation

**Figure 6:**
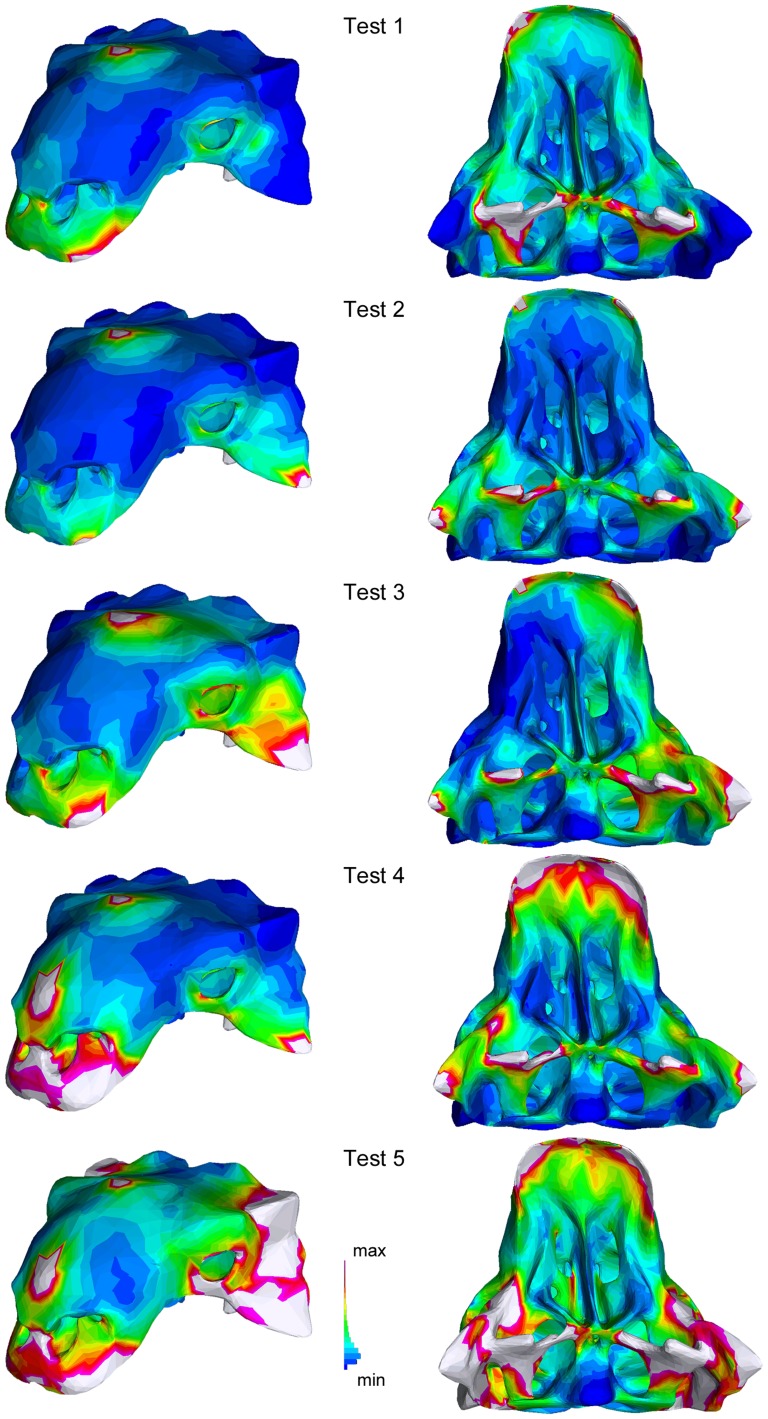
Results of the finite element analyses simulating taphonomic deformation in *Euoplocephalus*. AMNH 5405 in oblique rostrolateral view (left column) and ventral view (right column).

The five FEA tests progressively increase the number of constraints and force locations (Table 2), which results in progressively greater overall strain in the model. In Test 1 for AMNH 5405, strain is greatest at the premaxillae, jugals (and possibly lacrimals), vomers, palatines, pterygoids, paroccipital processes, and at the forces and constraints (Fig. 6). The addition of constraints at the quadratojugal horns in Test 2 decreased the strain at the premaxillae and the quadrate heads relative to Test 1, but increased the strain on the quadratojugal horns. The shearing force modeled in Test 3 resulted in an asymmetric strain distribution on the skull. Test 4 added a force on the nasal, and resulted in increased strain on the premaxilla and maxilla. The addition of forces at the squamosal horns in Test 5 resulted in increased strain on the frontals, prefrontals, parietals, squamosals, quadratojugals, and much of the ventral surface of the skull except for the occipital condyle.

**Figure 7:**
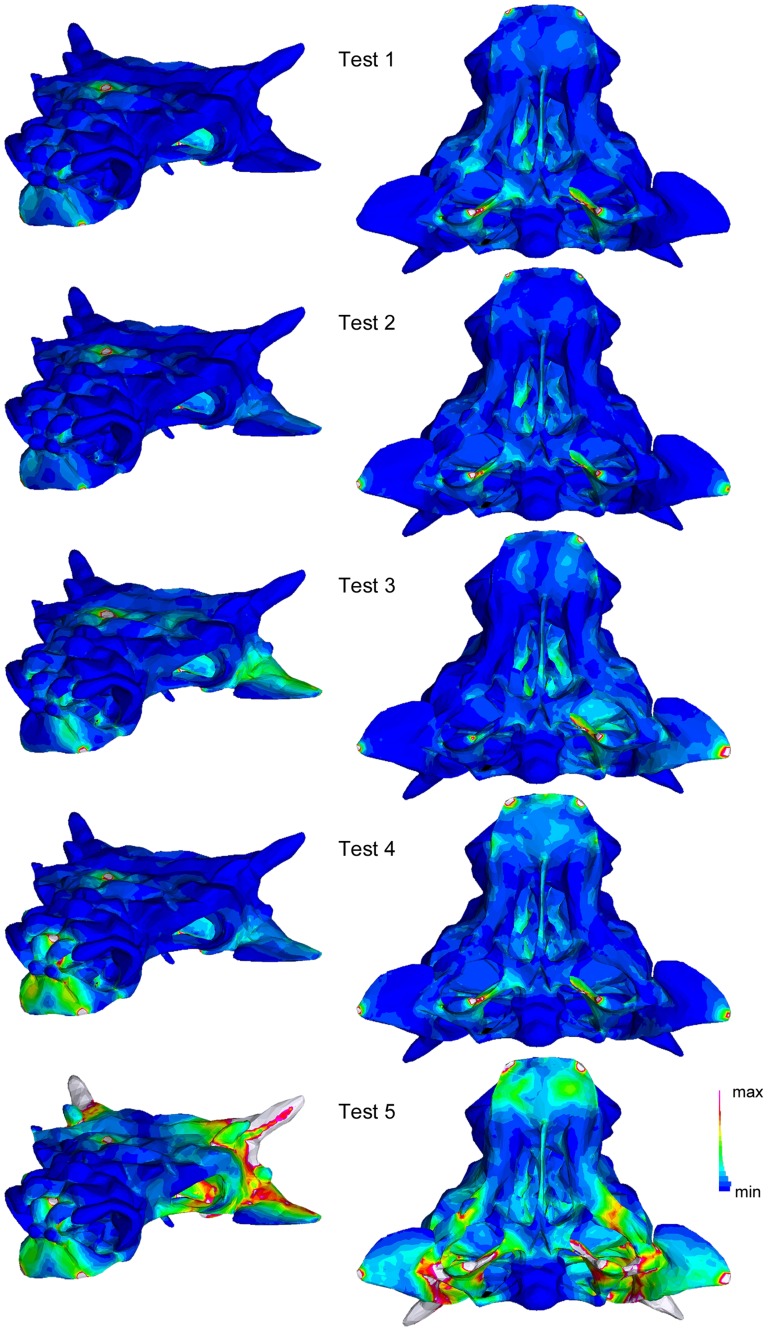
Results of the finite element analyses simulating taphonomic deformation in *Minotaurasaurus*. INBR 21004 in oblique rostrolateral view (left column) and ventral view (right column).

The FEA tests on INBR21004 were generally similar to that of AMNH 5405 (Fig. 7). In Test 1, strain was greatest on the jugals, quadrates, vomers, and palatines, and at the forces and constraints. In Test 2, where constraints were added to the quadratojugals, strain increased along the quadratojugals. Strain was asymmetrically distributed in Test 3. The addition of a force on the nasals in Test 4 resulted in increased strain on the premaxillae. Test 5 added forces to the squamosal horns, and resulted in increased strain on the premaxillae, jugals, lacrimals, quadratojugals, squamosals, quadrates, pterygoids, and paroccipital processes.

In both models, strain was high within and below the nares, but low on the narial osteoderms (Figs. 4, 5). The paroccipital processes experienced more strain in AMNH 5405 than in INBR21004. The distribution of strain around the orbit also differed between the two skulls: in AMNH 5405, strain was high in all of the bones surrounding the orbit, whereas in INBR21004 strain was high only on the bones forming the ventral border of the orbit.

Strain is artificially high at the constraints and nodes, and it is important to remember that in reality a skull experiencing taphonomic deformation would be crushed along more surfaces than are represented in the tests presented here. However, these tests indicate which areas of the skull were most likely to experience strain, and as a result were more likely to deform, relative to other areas of the skull.

## Discussion

Taphonomic distortion of some ankylosaur skulls is immediately easy to identify if there are obvious and extreme asymmetries, such as those seen in the holotypes of *Crichtonsaurus benxiensis* Lü, Ji, Gao, and Li, 2007 [31] (BXGM V0012) and *Nodocephalosaurus kirtlandensis* Sullivan, 1999 [32] (SMP VP900). Prieto-Márquez [33] noted that bending ridges and unusual bulges can also be signs of dorsoventral crushing in fossil skulls. However, Boyd and Motani [34] have shown that a symmetrical model does not indicate that plastic deformation from overburden compaction has been removed, and it can be easy to reconstruct a skull into an incorrect shape if there is no knowledge of accurate skull morphology. As such, symmetry alone may be insufficient for identifying deformation.

Measurements of the ellipticity of extant, undeformed vertebrate orbits suggest that orbits are not perfectly circular, but that the length:height ratio is generally between 1.00 and 1.28. As such, elliptical orbits in fossil specimens may not necessarily indicate that dorsoventral compaction has occurred. However, an orbit shape ratio greater than 1.28 in fossil skulls may indicate that some amount of dorsoventral crushing has occurred.

The higher orbit ratios in the few crocodilian and avian taxa in this study (representing the extant phylogenetic bracket for ankylosaurs) may suggest that archosaurian orbits are less circular than those of mammals, and that undeformed orbit ratios from 1.3–1.7 could be expected for dinosaurs. However, many of the ankylosaurid skulls had orbit ratios well above the maximum undeformed ratio recorded in this study (1.66 for *Varanus* sp.), and the range of orbit ratios was much greater for ankylosaurs than for all extant taxa combined. A plot of ankylosaur orbit ratios (Fig. 3) shows that few specimens have a ratio below 1.28. This suggests that either ankylosaurid orbits were not generally circular, or that many skulls have undergone some dorsoventral crushing during fossilization and diagenesis. AMNH 5405 has surprisingly high orbit ratios, given that the arched profile of the skull suggests little crushing took place. In contrast, *Crichtonsaurus* has a relatively low orbit ratio, despite the fact that this skull is highly asymmetrical and has certainly been flattened and distorted. Several specimens (AMNH 5403, MOR 433) have noticeably different orbit ratios for the left and right orbits, which suggests that the skulls underwent shearing or uneven dorsoventral compaction. Orbit ratios may be most useful when compared across multiple specimens of the same taxon, and very high ratios above 2 (in specimens where the orbit is completely encircled by the periorbital rim) are likely to indicate that dorsoventral crushing has occurred. The orbit ratio can serve as a general indicator if an ankylosaurid skull has been dorsoventrally compacted, but cannot be used to definitely indicate how much compaction has occurred. The true orbit ratio may not be known for a given fossil taxon, but high orbit ratios relative to the mean for a given sample of fossil specimens could also be used to identify if dorsoventral compaction has occurred. The orbit ratio could be a useful indicator of compaction for skulls that are symmetrical and which may not be obviously deformed.

Geomagic is a useful tool for investigating potential shape changes resulting from dorsoventral compression. The results of these tests can be independently assessed using finite element analysis to investigate which areas of the skull are most likely to experience strain (and therefore shape change). The FEA tests (Figs. 5, 6) showed high strain on the jugals, quadratojugals, and squamosals, which correspond to areas of change in the Geomagic models (Fig. 4). Strain was also present on the quadrates, pterygoids, and vomers, which did not change much in the Geomagic models. This indicates that retrodeforming a flattened skull in Geomagic will provide a good approximation for which features have been most affected, but may not reveal changes in all regions of the skull. Finite element analysis of several taphonomic scenarios is useful for determining which forces a skull may have been subjected to during deformation.

Taphonomic distortion may be responsible for some of the variation in skulls referred to *Euoplocephalus*. For example, Penkalski [19] suggested that the more upright squamosal horns of MOR 433 (in comparison to USNM 11892) may have been a result of crushing. This is supported by results from this study, where dorsoventrally compressing AMNH 5405 in Geomagic resulted in more upright squamosal horns similar to those of UALVP 31 (Fig. 4). The most noticeable change to AMNH 5405 was the flattening of the skull in lateral view. Skulls referred to *Euoplocephalus* have a range of morphologies in lateral view, from arched (AMNH 5405, ROM 1930), to flat (CMN 8530, USNM 11892). It is possible that the arching of the skull may be related to ontogeny, in which case a correlation between flatness and size would be expected. It is also possible that the relative flatness may be a true taxonomic difference. However, many of the skulls that are flat also have subcircular orbits, which suggests that the skulls have undergone crushing and in life were more arched.

Miles and Miles [12] identify several features of *Minotaurasaurus* as being flatter or more horizontal than their equivalents in other ankylosaurids: the angle of projection of the jugal horns, the articular surface of the quadrate, the pterygoid-quadrate contact, and the orientation of the pterygoid body. Additionally, the ‘flaring’ narial osteoderms may be a product of dorsoventral crushing. Retrodeformation of INBR21004 in Geomagic resulted in more ventrally projecting quadratojugal horns, but did not affect the quadrates or pterygoids (Fig. 4). However, finite element analyses simulating crushing in INBR21004 showed increased strain (and therefore shape change) in the quadrates and the caudal portion of the pterygoids (Fig. 6). This suggests that the retrodeformation techniques outlined in this study do not necessarily capture all of the shape changes on the ventral side of the skull, and emphasizes the need for multiple approaches when attempting to understand deformation in fossils. The dorsoventral angle of projection of the quadratojugal horn can be easily affected by taphonomic distortion, and should not be used as a diagnostic character for ankylosaur taxa. It is less clear if the articular surface of the quadrate, pterygoid-quadrate contact and horizontal pterygoid body in *Minotaurasaurus* are a result of deformation or represent true taxonomic differences. The flaring appearance of the narial osteoderms did not change during retrodeformation (Fig. 4), and dorsoventral compaction of AMNH 5405 did not result in more flaring narial osteoderms. UALVP 31, which is probably dorsoventrally compacted, also lacks flaring narial osteoderms (Fig. 4). In the finite element analyses of INBR21004, the narial osteoderms never experienced increased strain under any of the load regimes (Fig. 6). This suggests that the wide, flaring nares of *Minotaurasaurus* are real, and not an artifact of preservation.

Although Geomagic contains tools that could be used to correct plastic deformation in a fossil, there are many challenges associated with reconstructing a distorted fossil into its true, original shape. It is difficult to determine the accuracy of the retrodeformed skull in which there is no extant, undeformed analog. Simply restoring symmetry is insufficient to determine if a retrodeformed skull represents an accurate shape. Boyd and Motani [34] demonstrated that a digitally fragmented and distorted skull could be pieced back together into a symmetrical, but incorrect shape. As such, the results presented in this paper should not be taken to indicate that dorsoventrally compacted ankylosaur skulls can be retrodeformed into their true shape, but that retrodeformation tools can be used to understand which parts of the skull were most likely to be deformed. Three-dimensional retrodeformation techniques are useful for understanding potential sources of morphological variation in ankylosaur skulls, but it is not possible to confidently retrodeform an ankylosaur skull to its original shape.

Retrodeformation of a specimen may result in new taxonomic interpretations because of changes in shape. The accuracy of 3D retrodeformation techniques is still being investigated; retrodeformation is more likely to be successful when morphological constraints, based on features of extant taxa, can be used [3]. Although the FEA results differed somewhat from the retrodeformation results, some morphological features consistently changed (or did not change), and this provides information on which ankylosaur cranial characters may or may not be taxonomically informative. Overall skull morphology was easily changed with minimal retrodeformation, but features of the palate and braincase were less likely to be affected. The dorsoventral angle of projection of the quadratojugal horn is easily altered by dorsoventral compaction and should not be used to support taxonomic distinctions among ankylosaurs. Many of the diagnostic features of *Minotaurasaurus* did not change during retrodeformation, which suggests that these features are either unique to this genus or represent intraspecific or ontogenetic variation within a different taxon. Much of the variation in skull morphology in specimens referred to *Euoplocephalus* may also be a result of taphonomic distortion, although again intraspecific and ontogenetic variation cannot be ruled out.
